# Fibroblast stimulation of breast cancer cell growth in a serum-free system.

**DOI:** 10.1038/bjc.1993.237

**Published:** 1993-06

**Authors:** M. C. Ryan, D. J. Orr, K. Horgan

**Affiliations:** Department of Surgery, University of Wales College of Medicine, Heath Park, Cardiff, UK.

## Abstract

Conditioned media from 14 short term fibroblast cell lines were mitogenic for human breast cancer cells with different steroid receptor profiles in serum-free culture. Fibroblast-conditioned medium stimulated tritiated thymidine incorporation in short term culture and growth in a longer proliferation study as measured by the MTT colorimetric assay. Conditioned media from benign and malignant epithelial cells were non-stimulatory for breast cancer cells but that derived from endothelial cells showed similar stimulation to fibroblasts. Partial purification of fibroblast-conditioned medium identified a peptide with a molecular weight of approximately 8 kDa that showed no affinity for heparin and was mitogenic for MCF-7 breast cancer cells.


Br. J. Cancer (1993), 67, 1268-1273 © Macmillan Press Ltd., 1993

### Fibroblast stimulation of breast cancer cell growth in a serum-free system

M.C. Ryan, D.J.A. Orr & K. Horgan

*Department of Surgery, University of Wales College of Medicine, Heath Park, Cardiff CF4 4XN, UK.*

**Summary** Conditioned media from 14 short term fibroblast cell lines were mitogenic for human breast cancer cells with different steroid receptor profiles in serum-free culture. Fibroblast-conditioned medium stimulated tritiated thymidine incorporation in short term culture and growth in a longer proliferation study as measured by the MTT colourimetric assay.

Conditioned media from benign and malignant epithelial cells were non-stimulatory for breast cancer cells but that derived from endothelial cells showed similar stimulation to fibroblasts.

Partial purification of fibroblast-conditioned medium identified a peptide with a molecular weight of approximately 8 kDa that showed no affinity for heparin and was mitogenic for MCF-7 breast cancer cells.

There is evidence for autocrine control of human breast cancer growth, through endogenous or oestrogen controlled production of polypeptide growth factors (Lippman *et al.*, 1986*a*; 1986*b*; Cullen *et al.*, 1989). It has also been proposed that paracrine interactions between stromal tissue and malignant epithelia play an important role in the development and control of human breast cancer (Lippman *et al.*, 1986*a*; 1986*b*; Cullen *et al.*, 1989). Human breast cancer cells have been shown to express growth factors for mesenchymal cells (Rozengurt *et al.*, 1985; Peres *et al.*, 1987), suggesting a molecular basis for the generation of tumour stroma. There is controversy however as to whether neighbouring stromal tissue cells secrete soluble factors that regulate the growth of malignant epithelia (Yee *et al.*, 1988; 1989; Cullen *et al.*, 1991; Clarke *et al.*, 1992). Although studies have demonstrated that fibroblasts increased the growth of human breast cancer cells, the evidence remains inconclusive as to the extent and nature of the paracrine stimulation.

This laboratory (Horgan *et al.*, 1987) and others (Camps *et al.*, 1990) have reported that co-inoculation of human breast cancer cells and fibroblasts increased both the 'take' and size of xenotransplants in athymic mice. Fibroblasts that had been pre-treated with glutaraldehyde increased MCF-7 tumour take but not growth (Horgan *et al.*, 1987), whereas lethally irradiated fibroblasts (Picard *et al.*, 1986; Camps *et al.*, 1990) and fibroblast-conditioned medium (Picard *et al.*, 1986) stimulated tumour growth, though to a lesser extent than live fibroblasts. This suggests that tumour take is induced by an inert fibroblast factor, whereas maximal growth stimulation of malignant epithelia required the continuous supply of a tumour promoting factor by the interacting fibroblasts.

Studies of the influence of breast fibroblasts on the growth of breast cancer cells *in vitro*, have provided conflicting results. Mukaida *et al.* (1991) demonstrated that fibroblasts from mammary gland tissue increased the growth of MCF-7 cells when co-cultured in double layer soft agar. However, conditioned medium from these fibroblasts failed to stimulate MCF-7 cell growth in both anchorage independent and anchorage dependent growth assays. Adams *et al.* (1988*a*) demonstrated that conditioned medium from fibroblasts derived from malignant and benign breast tumours increased the growth of the human breast cancer cell line MCF-7 *in vitro*. As they used a serum based culture system, interaction between growth factors in the serum and proteins in the conditioned medium may be responsible for the stimulation in breast cancer cell growth. This laboratory (Ryan *et al.*, 1991) and others (Van Roozendaal *et al.*, 1992) have demonstrated that fibroblast-conditioned medium stimulates breast cancer cell growth in serum free culture, indicating the production of a mitogenic factor by human fibroblasts.

In this paper, we investigate the importance of fibroblast derived products in paracrine stimulation of human breast cancer cells *in vitro* using a serum free assay system. We compare the mitogenic activity of fibroblast-conditioned medium with that of both benign and malignant epithelia and endothelium. Finally, we investigate the characteristics of the factor or factors present in fibroblast-conditioned medium that are mitogenic for human breast cancer cells *in vitro*.

### Materials and methods

All chemicals were supplied by Sigma Chemical Co, Dorset, UK. Except where specifically noted.

### *Tissue culture media*

The basic tissue culture media used throughout was DMEM/F12 1:1(phenol red free) supplemented with 15 mM HEPES, 2.2 g $l^{-1}$ sodium bicarbonate and the antibiotics Benzylpenicillin (Glaxo Lab Ltd, Greenford UK) 50 units $ml^{-1}$, Streptomycin (Evans Medical Ltd, Greenford UK) 100 $\mu g$ $ml^{-1}$ and Amphotericin B (Squibb, Hounslow, UK) 2 $\mu g$ $ml^{-1}$. This is referred to as serum free medium (SFM).

For routine maintenance of fibroblasts and the breast cancer cell lines SFM was supplemented with human monocomponent insulin 0.12 iu $ml^{-1}$ (Novo Industries, Romford, UK), hydrocortisone 4 $\mu g$ $ml^{-1}$ (Upjohn Ltd, Crawley, UK) and 10% Fetal Calf Serum (FCS) (Gibco Ltd, Paisley, UK) referred to as complete medium (CM). Non malignant breast epithelia were maintained in CM further supplemented with cholera toxin (10 ng $ml^{-1}$) and epidermal growth factor (EGF) (10 ng $ml^{-1}$).

The malignant breast cell lines were grown in basal medium containing dextran/charcoal stripped 10% FCS (DCC-CM) for 48 h prior to growth assays. The cells were grown in serum free medium supplemented with BSA (200 $\mu g$ $ml^{-1}$) and transferrin (10 $\mu g$ $ml^{-1}$) (SFM-BSA/Tf) during proliferation assays.

### *Primary cultures of breast fibroblasts and epithelia*

Samples of breast tissue and a wound biopsy were dissected free of fat and cut into small (1-5 mm) pieces.

The dissected tissue was subjected to collagenase digestion (0.5 mg $ml^{-1}$ collagenase) and the samples mixed overnight on a blood wheel at 37°C. The digest was centrifuged, 5 ml of fresh media added and allowed to sediment under gravity for 20 min at room temperature. The initial supernatant was plated into tissue culture flasks as a fibroblast-rich suspension. The sedimentation process was repeated and subsequent supernatants and the final cell sediment were plated as

Correspondence: K. Horgan.
Received 10 November 1992; and in revised form 13 January 1993.

sources of mixed fibroblast/epithelial cultures. After 5–7 days incubation, when epithelial colonies were well established, the cultures were differentially trypsinised to detach fibroblasts from the flask while leaving the epithelial cells attached. Fourteen fibroblast cell lines were established: four from fibroadenoma tissue, four from normal breast parenchyma adjacent to benign and malignant breast disease, three from malignant breast tissue, two from breast skin tissue and one from a granulating wound. Fibroblasts only survived in short term culture and were used between passages two and 12. They did not form tumours when injected into nude mice, and demonstrated typical fibroblast morphology in culture. The fibroblast cultures were confirmed as fibroblasts by staining with the monoclonal antibody Dako-Fibroblast 5B5 (Dako Ltd, High Wycombe, UK) which reacts with the β-subunit of prolyl-4-hydroxylase and with the disulphide isomerase.

*Breast tumour cell lines*

Five breast cancer cell lines were utilised: MCF-7, T47D, MDA-MB-231, MDA-MB-436, and ZR-751. MCF-7, T47D and MDA-MB-436 were obtained as generous gifts from Dr Colby Eton (Tenovus Cancer Research Institute, Cardiff UK). MDA-MB-231 and ZR-751 from Dr Marc Lippman (NIH, Bethseda, Maryland, USA) and Dr W.R. Miller (Imperial Cancer Research Fund, Medical Oncology Unit, Western General Hospital, Edinburgh, UK) respectively.

*Endothelial cells*

Primary endothelial cells (MENDO 1) that had been isolated from a human umbilical vein and grown in short term culture were supplied by Mr Simon Evans (Dept. Haematology, UWCM, Cardiff, UK). The endothelial cultures were demonstrated to contain greater than 95% cells that stained positive for Von Willebrand's factor using a monoclonal antibody Dako-factor VIII (Dako Ltd, High Wyecombe, UK).

*Preparation of conditioned medium*

Cells were grown in CM until 75–90% confluency, washed twice with SFM and grown in SFM for 24 h. For the purposes of comparison between different fibroblasts, identical numbers of cells ($10^6$) were used to produce 15 ml of conditioned medium. The conditioned medium was collected and centrifuged at 3000 RPM in a bench top centrifuge for 15 min and filtered through a 0.2 μm filter. The filtrate was stored at − 20°C until required. Forty-eight hours prior to the production of epithelial cell conditioned medium cholera toxin and EGF were removed from the medium.

*Growth assay*

Breast tumour cell lines were plated into 96-well microtitre plates (Nunc, Denmark) at a concentration of 5000 cells (1,250 cells MDA-MB-231) per well in SFM containing 5% DCC-FCS. After 24 h the plates were washed twice with SFM and varying dilutions of fibroblast, epithelial or endothelial cell conditioned medium or concentrations of known growth factors were added in SFM-BSA/Tf. The concentrations of BSA and transferrin were maintained constant in all experiments. The cultures were incubated for between 1 and 7 days and the effects on growth measured.

*MTT assay*

Cell proliferation was measured using a colourimetric assay (Mossman, 1983) based on the ability of viable cells to convert a soluble tetrazolium salt, 3,[4,5-Dimethylthiazol-2-yl]-2,5,-diphenyltetrazolium bromide (MTT) into an insoluble formazan precipitate. Following initial calibration experiments, the assay was modified from the procedure of Twentyman and Luscombe (1987). Twenty μl MTT (2.5 mg $ml^{-1}$ Phosphate Buffered Saline pH 7.2) was added for 4 h and all medium was removed leaving purple crystals of insoluble tetrazolium at the bottom of the wells. The crystals were redissolved by the addition of 200 μl DMSO and the absorbance was measured in a Titertek multiwell spectrophotometer at 550 nm.

*Tritiated thymidine assay*

Cells were grown for 20 h, following which, $^3$[H]-thymidine (Amersham UK plc) was diluted in thymidine containing medium and added to each well at a final concentration of 2 μci $ml^{-1}$ for a period of 8 h. Subsequently the medium was removed, the cells incubated for 15 min in ice cold 10% TCA, washed three times in 5% TCA for 5 min, lysed overnight in 0.5 M NaOH and the incorporated radioactivity quantified by scintillation counting.

*Characterisation of the mitogenic component in fibroblast-conditioned medium*

*Heat stability* Conditioned medium was heated to 56°C and 100°C for 30 min and the stimulatory activity tested on MCF-7 cells.

*Trypsin sensitivity*

Trypsin at a concentration of 10 μg $ml^{-1}$ was added to fibroblast-conditioned medium and incubated for 1 h at 37°C. The effect of the trypsin was neutralised by the addition of soyabean trypsin inhibitor at a concentration of 20 μg $ml^{-1}$. As a control conditioned medium was incubated for 1 h with trypsin 10 μg $ml^{-1}$ together with trypsin inhibitor, 20 μg $ml^{-1}$ at 37°C. Recovery of mitogenic activity following trypsin exposure was calculated as a percentage of this control.

*Stability to reducing agents*

Dithiothreitol was added to fibroblast-conditioned medium at a final concentration of 50 mM for 1 h at room temperature, the medium was then dialysed at 4°C against three changes of SFM using Spectra/Por 3 dialysis tubing (Spectrum Medical Industries, Inc, USA) and mitogenic activity assayed.

*Acid and ionic stability*

Fibroblast-conditioned medium was dialysed for 4 h against 1 M acetic acid and distilled water at 4°C using Spectra/Por 3 dialysis tubing (mol wt cut off. 3,500) (Spectrum Medical Industries, Inc, USA). The pH of acid treated medium was demonstrated to have fallen to pH 2.0. The medium was then dialysed against SFM until the pH of the acid treated medium had returned to 7.3. Mitogenic activity was then assayed. As a control, fibroblast-conditioned medium was dialysed for 4 h against SFM and the activity recovered following dialysis against acid and distilled water calculated as a percentage of this control.

*Heparin binding*

Twenty-five ml of fibroblast-conditioned medium was added to a 5 ml Hi-trap Heparin (Pharmacia-LKB Sweden) affinity column. The unabsorbed material was collected and the mitogenic activity compared to that of untreated conditioned medium. The column was then eluted with increasing concentrations of NaCl in 20 mM tris buffer pH 7.2. Fractions were collected and dialysed against three changes of SFM using Spectra/Por 3 dialysis tubing (Spectrum Medical Industries, Inc, USA). The mitogenic activity of each fraction was assayed.

*Sephadex G-75 column*

Fibroblast-conditioned medium was purified using a modification of the procedure described by Adams *et al.* (1988*b*). Fibroblast-conditioned medium was concentrated 10 fold by ultrafiltration using a YM2 filter (mol wt cut off 1000) (Amicon Ltd, UK). The concentrate was then added to a Sephadex G-75 column (1.65 cm × 65 cm). The sample was eluted from the column under gravity with 250 ml PBS (pH 7.4) at 4°C. Fractions (4 ml) were collected, sterilised by passage through a 0.2 μm Millipore filter and tested immediately at a 20% v/v concentration.

The Mann-Whitney test was used for statistical analysis.

### Results

### *Effect of fibroblast-conditioned medium on MCF-7 cells in serum free culture*

MCF-7 cells grown in SFM-BSA/Tf grow slowly but remain viable. Addition of fibroblast-conditioned medium stimulated growth in a dose-dependent manner (Figure 1), with 50% conditioned medium producing a 119% increase over cells grown in SFM-BSA/Tf at day 7 ($P<0.01$). Fibroblast-conditioned medium increased cell growth to 90% of that produced by cells grown in complete medium containing 5% FCS.

### *Comparison of growth assays*

To prove that the increase in MTT absorbance reflected a genuine increase in cell number rather than an increase in cell respiration, we compared the effect of fibroblast-conditioned medium on MTT absorbance after 6 days in culture and on $^3$[H]-thymidine uptake after 28 h (Figure 2). The fibroblast-conditioned medium increased $^3$[H]-thymidine uptake and MTT absorbance in a concentration dependent manner. At a 50% dilution in SFM (BSA/Tf), conditioned medium produced an increase in $^3$[H]-thymidine of 132.5% ($P<0.01$) an increase in MTT absorbance of 102.2% ($P<0.01$) over cells grown in SFM (BSA/Tf).

### *Effect of fibroblast-conditioned medium from different cell lines*

To determine that growth stimulation was not a characteristic of one particular fibroblast cell line, the effect of 14 different fibroblast conditioned media on MCF-7 cell growth was measured. The results are presented in Table I. All tested fibroblasts produced conditioned medium that was stimulatory for MCF-7 cell growth in serum free medium independent of tissue source. The degree of stimulation varied from 31.3% to 123.4% increase in MCF-7 cell growth. The numbers of cell lines in each group are too few to discern any differences in the degree of stimulation induced by fibroblasts of different tissue origin.

**Figure 2** Comparison of growth assays for BBF-10 fibroblast stimulation of MCF-7 cells. MTT (●-●) colourimetric assay after 6 days growth, 3[H]-thymidine uptake (O-O) after 28 h in culture. Results shown as mean ± standard deviation ($n = 6$).

**Table I** Comparison of mitogenicity for MCF-7 cells of conditioned medium from fibroblasts of different tissue sources

| *Fibroblast line* | *Tissue source* | *% Stimulation ± s.e.m.* |
|---|---|---|
| KB16F | Fibroadenoma | 73.3% ± 8.9% |
| DB10F | Fibroadenoma | 119.8% ± 7% |
| MB1F | Fibroadenoma | 68.2% ± 1.8% |
| MB2F | Fibroadenoma | 31.8% ± 2.6% |
| MB3F | Normal breast | 123.4% ± 5% |
| BBF1 | Normal breast | 98% ± 5.4% |
| BBF5 | Normal breast | 83.5% ± 6.5% |
| BBF10 | Normal breast | 121.9% ± 3.7% |
| BTF1 | Malignant breast | 114% ± 5.3% |
| BTF3 | Malignant breast | 48.1% ± 5.6% |
| BTF9 | Malignant breast | 66.8% ± 4.6% |
| SF1 | Skin | 99.2% ± 6.2% |
| SF2 | Skin | 60.7% ± 2.9% |
| WF2 | Granulating wound | 31.3% ± 4.3% |

**Figure 1** Response of MCF-7 cells to BBF-10 fibroblast-conditioned medium. Growth in SFM (□-□), in CM (O-O), in SFM-BSA/Tf (■-■), in 25% (▲-▲), in 50% (▼-▼) and 100% (●-●) fibroblast-conditioned medium, as measured by the MTT assay. Results are shown as mean ± standard error.

### *Effect of fibroblast-conditioned medium on a number of breast cancer cell lines*

The stimulatory effects of fibroblast-conditioned medium for a number of different breast cancer cell lines in serum free medium, are shown in Figure 3. The results are presented as increase in DPM and MTT absorbance over cells grown in SFM (BSA/Tf), as the individual cell lines grow at different rates in this medium. The fibroblast-conditioned medium was stimulatory for all the tested breast cell lines. $^3$[H]-Thymidine was increased by 132.5% in MCF-7 cells, 135.4% in T47D cells, 111.9% in MDA-MB-231 cells, 107.1% in MDA-MB-436 cells and 102.6% in ZR-751 cells at a dilution of fibroblast-conditioned medium of 50% in SFM (BSA/Tf) ($P<0.01$) (Figure 3a). Fibroblast-conditioned medium at a concentration of 50% increased MTT absorbance after 6 days in culture, for MCF-7, T47D, MDA-MB-231, MDA-MB-436 and ZR-751 by 53.9%, 59.5%, 66.6%, 48.8% and 37.1% respectively [$P<0.01$] (Figure 3b).

**Figure 3** Effect of BBF-10 fibroblast-conditioned medium on the growth of five breast cancer cell lines **a**, Effect on 3[H]-thymidine uptake after 28 h in culture; **b**, Effect on MTT absorbance after 6 days in culture. MCF-7 (●-●), T47D (□-□), MDA-MB-231 (◆-◆), MDA-MB-436 (▽-▽), ZR.75-1 (△-△) Results presented as means ± standard error.

*Comparison of conditioned medium from breast fibroblasts, benign breast epithelia, malignant breast epithelia, and endothelia*

Undiluted benign epithelial conditioned medium inhibited MCF-7 cell growth (28.9% $P<0.01$), however dilution to 50% in SFM (BSA/TF) produced a small but significant increase in MCF-7 cell growth with a 22.7% increase in MTT absorbance ($P<0.01$) (Figure 4). Malignant epithelial conditioned medium was not mitogenic, in undiluted form producing a significant inhibition of MCF-7 cells (11.8% $P<0.05$) (Figure 4). Similar results with malignant epithelial conditioned medium have been reported (Van der Burg *et al.*, 1990). Endothelial cells were highly stimulatory, their conditioned medium causing significant increases in MTT absorbance, 98.4% ($P<0.01$) at a dilution of 50% in SFM (BSA/Tf) (Figure 4). Endothelial cells were found to be the only other cell type, beside fibroblasts to stimulate the growth of human tumours as xenotransplants in athymic mice (Picard *et al.*, 1986) suggesting that mesenchymal cells produce mitogenic factors for human tumour cells.

*The effect of polypeptide growth factors on MCF-7 cells*

The effects of a number of polypeptide growth factors on the growth of MCF-7 breast cancer cells *in vitro* were determined (Figure 5).

EGF was a potent mitogen at a concentration of 10 ng $ml^{-1}$, increasing MTT absorbance by 116.3% over control cells. Insulin-like growth factors were also mitogenic, in the order of potency IGF1 > IGF2 > insulin increasing cell growth by 131.7%, 75.2%, and 19% respectively. PDGF and TGF-$\beta$ induced no significant increase in growth. These

**Figure 4** Comparison of the effect of conditioned medium from fibroblast, endothelial, benign breast epithelial and malignant breast epithelial cell lines on the growth of MCF-7 breast cancer cells *in vitro*. MB3F fibroblast-conditioned medium (●-●), Mendo 1 endothelial-conditioned medium (▲-▲), MB3E benign epithelium-conditioned medium (■-■), T47D malignant epithelium-conditioned medium (◆-◆). Growth measured by the MTT colourimetric assay after 6 days in culture, results presented as mean % increase in absorbance at 550 nm ± standard error ($n = 6$).

**Figure 5** Effect of polypeptide growth factors on the growth of MCF-7 breast cancer cells *in vitro* as measured by the MTT colourimetric assay after 6 days in culture. Growth in SFM-BSA/Tf (□), in SFM-BSA/Tf plus 10 ng $ml^{-1}$ polypeptide growth factor (■). Results presented as mean ± s.e. ($n = 6$). $^{**}P<0.01$, $^{*}P<0.05$, n.s. = not significant.

results concur with the report of Karey and Sirbasku (1990) and confirm that the effects of known mitogens were detectable in our assay system.

*Characterisation of the mitogenic component of fibroblast-conditioned medium*

The effect of heat, trypsin, dithiothreitol, acid and ionic treatment on the mitogenic activity of fibroblast-conditioned medium is presented in Table II.

The mitogenic component of fibroblast-conditioned medium is relatively heat and acid stable with exposure to 100°C and 1 M acetic acid reducing the activity to 38% and 48% of control levels respectively, whereas dialysis against distilled water reduces the mitogenic activity to 22%. The protein nature of the mitogenic component is confirmed by the reduction in activity to 55% following exposure to trypsin and total loss of activity following dithiothreitol treatment.

Fibroblast-conditioned medium that had been passed through a heparin column was as stimulatory as untreated conditioned medium, and the fractions collected following elution with increasing concentrations of NaCl were found to contain no stimulatory activity.

Fibroblast-conditioned medium was subjected to a crude purification on a Sephadex G-75 column. The effect of individual fractions on the growth of MCF-7 cells in SFM (BSA/Tf) is presented as MTT absorbance (Figure 6). The values for fractions obtained from conditioned medium are shown alongside values obtained from purification of SFM. In three separate fractionations a peak of increased cell growth was detectable at fraction 14, which corresponds to a molecular weight of 8,000 Da approximately.

**Table II** Physico-chemical characteristics of the mitogenic component of fibroblast conditioned medium

| *Treatment* | *% activity recovered* |
|---|---|
| 56°C for 30 min | 79.2% |
| 100°C for 30 min | 38.3% |
| Trypsin 10 $\mu$g ml$^{-1}$ 1 h 37°C | 55% |
| 50 mM Dithiothreitol for 1 h | 0% |
| Acid dialysis to pH 2 | 48% |
| Dialysis against distilled $H_2O$ | 22% |

**Figure 6** Fractionation of KB16F fibroblast-conditioned medium on a Sephadex G-75 column. Effect of individual fractions on the growth of MCF-7 cells. Growth was measured after 6 days in culture by the MTT colourimetric assay. Fractions of fibroblast-conditioned medium (●-●), molecular weight markers (◆-◆) (Haemoglobin, Cytochrome C, Dinitrophenol). This profile is the mean of three separate experiments.

### Discussion

The results demonstrate that conditioned media from short term cultures of human fibroblasts are mitogenic for a number of human breast cancer cell lines, increasing both DNA synthesis in short term culture and cell number in a more long term proliferation assay. These findings support the concept that stromal cells may play an important role in the regulation of human breast tumour growth, through the production of factors that modulate the proliferation of tumour epithelia (Lippman *et al.*, 1986*a*; 1986*b*; Yee *et al.*, 1988; 1989; Cullen *et al.*, 1991).

Van Roozendaal *et al.* (1992), using a serum free culture system and the MTT proliferation assay, demonstrated that breast tumour cells responded differently to fibroblast-conditioned medium: the steroid receptor positive cell lines MCF-7 and ZR 75.1 were stimulated to grow, whereas the steroid receptor negative cell lines MDA-MB-231 and Evsa-T showed little or no proliferative response. We have demonstrated that fibroblast-conditioned medium increased radioactive thymidine uptake and MTT absorbance in all tested breast cancer cells.

Although it has been demonstrated that fibroblast stimulation of human breast cancer cell growth *in vivo* is independent of tissue source (Horgan *et al.*, 1987), Mukaida *et al.* (1991) reported that only fibroblasts from human mammary tissue and lung stimulated MCF-7 cell growth in a double layer soft agar assay. Fibroblasts from other tissues had no effect or were inhibitory. Adams *et al.* (1988*a*) reported that fibroblasts derived from malignant and benign breast tumours produced conditioned medium that stimulated MCF-7 cell growth *in vitro*, whereas fibroblasts derived from reduction mammoplasty tissue were inhibitory. Van Roozendaal *et al.* (1992) found that all fibroblasts, regardless of tissue source, stimulated MCF-7 growth *in vitro*. Our data concurs with this finding. However, they reported that the extent of the proliferative response was dependent on the source of the isolated fibroblasts. Tumour and skin fibroblasts induced a greater mitogenic effect on MCF-7 cells than those isolated from benign breast tissue.

Comparison of conditioned medium from fibroblast, benign epithelial, malignant epithelial and endothelial cell cultures showed that only cells derived from mesenchymal tissues were mitogenic for MCF-7 cells.

There is little information in the literature as to the nature and identity of the factors present in fibroblast-conditioned medium that stimulate the proliferation of tumour epithelia. Characterisation of the mitogenic component of fibroblast-conditioned medium demonstrates that after boiling for 30 min a residual activity of 30% remains and reduction to pH 2.0 reduces activity by half. The protein nature of the mitogenic component is suggested by the fact that it is sensitive to dithiothreitol and proteolytic enzyme treatment. The mitogenic component of fibroblast-conditioned medium has no affinity for heparin, therefore heparin binding growth factors such as basic and acidic fibroblast growth factors can be ruled out as the mitogenic factor in the conditioned medium. Partial purification of fibroblast-conditioned medium demonstrated that the cells secrete a mitogenic factor for MCF-7 cells with a molecular weight of approximately 8,000. TGF-$\alpha$ and the insulin like growth factors are small polypeptides with molecular weights similar to the factor identified and both factors have been shown by ourselves and others to be potent mitogens for MCF-7 cells *in vitro* (Karey & Sirbasku 1988; Van der Burg *et al.*, 1990; Osborne *et al.*, 1989, 1990). However, at the neutral pH at which the fibroblast-conditioned medium was extracted from the Sephadex column, IGFs would be expected to be bound to high affinity binding proteins and would elute at a much higher molecular weight. Normally, acid extraction would be required to extract free IGFs (Hintz, 1984).

IGF-I and IGF-II have been shown to be produced by human fibroblasts *in vitro* (Clemmons, 1984). *In situ* hybridisation studies (Yee *et al.*, 1989) have shown that IGF-I mRNA is expressed in the stromal tissue of breast tumours but not in the epithelial cells. Cullen *et al.* 1991 have demonstrated that short term cultures of fibroblasts isolated from benign tumours express high levels of IGF-I mRNA whereas fibroblasts isolated from malignant tumours express high levels of IGF-II mRNA. They also examined a panel of breast tumour cell lines (containing both oestrogen receptor positive and negative phenotypes) and breast cancer specimens. Messenger RNA encoding for IGF-I, IGF-II and Insulin receptors was present in virtually all samples tested (Cullen *et al.*, 1990). It has been suggested that the IGFs function as stromal derived factors that play an important role in the paracrine regulation of human breast cancer (Yee *et al.*, 1988; 1989; Cullen *et al.*, 1991). Clarke *et al.* (1992) dispute this hypothesis. While acknowledging that stromal fibroblasts secrete IGFs, they suggest that the IGFs act as autocrine factors inducing increased fibroblast growth. They point out that IGF production must be sufficient both to saturate the IGF receptors on stromal cells and to avoid inactivation by tumour produced inhibitory insulin binding proteins (Clemmons *et al.*, 1990).

The experiments detailed in this paper clearly demonstrate that fibroblasts secrete factors that stimulate human breast cancer *in vitro*. These factors may play an important role in the paracrine regulation of human breast cancer by stromal cells. Partial purification of the fibroblast-conditioned medium suggests that they secrete a non-heparin-binding peptide with a molecular weight very close to that of the IGFs and TGF-α. Further study is required to characterise or isolate fibroblast derived growth factors and to determine their role in epithelial-stromal interaction in the human breast.

We gratefully acknowledge the support for this work of Scotia Pharmaceuticals Ltd, Guildford, UK. We would also like to thank Mr Derek Jones for his help and advice with tissue culture.

### References

ADAMS, E.F., NEWTON, C.J., BRAUNSBERG, H., SHAIKH, N., GHILCHIK, M. & JAMES, V.H.T. (1988*a*). Effects of human breast fibroblasts on growth and 17β-estradiol dehydrogenase activity of MCF-7 cells in culture. *Breast Canc. Res. Treat.*, **11**, 165–172.

ADAMS, E.F., NEWTON, C.J., TAIT, G.H., BRAUNSBERG, H., REED, M.J. & JAMES, V.H.T. (1988*b*). Paracrine influence of human breast stromal fibroblasts on breast epithelial cells: secretion of a polypeptide which stimulates reductive 17β-Oestradiol dehydrogenase activity. *Int. J. Cancer*, **42**, 119–122.

CAMPS, J.L., CHANG, S.-M., HSU, T.C., FREEMAN, M.R., HONG, S.-J., ZHAN, H.E., VAN ESCHERBACK, A.C. & CHUNG, L.W.K. (1990). Fibroblast mediated acceleration of human epithelial tumor growth in vivo. *Proc. Natl Acad. Sci. USA*, **87**, 75–79.

CLARKE, R., DICKSON, R.B. & LIPPMAN, M.E. (1992). Hormonal aspects of breast cancer growth factors, drugs and stromal interactions. *Crit. Rev. Oncol/Hem.*, **12**, 1–23.

CLEMMONS, D.R. (1984). Multiple hormones stimulate the production of somatomedin by cultured human fibroblasts. *J. Clin. Endocrinol. Metab.*, **58**, 850–856.

CLEMMONS, D.R., CAMACHO-HUBNER, C., CORONADA, E. & OSBORNE, C.K.J. (1990). Insulin-like growth factor binding protein secretion by breast carcinoma cell lines: correlation with estrogen receptor status. *Endocrinology*, **127**, 2679–2686.

CULLEN, K.J., SMITH, H.S., HILL, S., ROSEN, N. & LIPPMAN, M.E. (1991). Growth factor messenger RNA expression by human breast fibroblasts from benign and malignant lesions. *Cancer Res.*, **51**, 4798–4985.

CULLEN, K.J., YEE, D., BATES, S.E., BRUNNER, N., CLARKE, R., DICKSON, R.E., HUFF, K.K., PAIK, S., ROSEN, N., VALVERIUS, E., ZUGMAIER, G. & LIPPMAN, M.E. (1989). Regulation of Human breast cancer by secreted growth factors. *Acta Oncol.*, **28**, 835–839.

CULLEN, K.J., YEE, D., SLY, W.S., PERDUE, J., HAMPTON, B., LIPPMAN, M.E. & ROSEN, N. (1990). Insulin-like growth factor expression and function in human breast cancer. *Cancer Res.*, **50**, 48–53.

HINTZ, R.L. (1984). Plasma forms of somatomedin and the binding protein phenomenon. *Clin. Endocrinol. Metab.*, **13**, 31–42.

HORGAN, K., JONES, D.L. & MANSEL, R.E. (1987). Mitogenicity of human fibroblasts *in vivo* for human breast cancer cells. *Br. J. Surg.*, **74**, 227–229.

KAREY, K.P. & SIRBASKU, D.A. (1988). Differential responsiveness of human breast cancer cell lines MCF-7 and T47D to growth factors and 17β-estradiol. *Cancer Res.*, **48**, 4083–4092.

LIPPMAN, M.E., DICKSON, R.B., BATES, S. & 7 others (1986*a*). Autocrine and paracrine growth regulation of human breast cancer. *Breast Canc. Res. Treat.*, **7**, 59–70.

LIPPMAN, M.E., DICKSON, R.B., KASID, A., GELMANN, E., DAVIDSON, N., MCMANAWAY, M., HUFF, K., BRANZERT, D., BATES, S., SWAIN, S. & KNABBE, C. (1986*b*). Autocrine and paracrine growth regulation of human breast cancer. *J. Steroid Biochem.*, **2**, 147–154.

MOSSMAN, T. (1983). Rapid colorimetric assay for cellular growth and survival: application to proliferation and cytotoxicity assays. *J. Immunol. Meth*,. **65**, 55–65.

MUKAIDA, H, HIRABAYASHI, N., HIRAI, T., IWATA, T., SAEKI, S. & TOGE, T. (1991). Significance of freshly cultured fibroblasts from different tissues in promoting cancer cell growth. *Int. J. Cancer*, **48**, 423–427.

OSBORNE, C.K., CLEMMONS, D.R. & ARTEAGA, C.L. (1990). Regulation of breast cancer growth by insulin-like growth factors. *J. Steroid. Biochem. Molec. Biol.*, **37**, 805–809.

OSBORNE, C.K., CORONADA, E.B., KITTEN, L.J. & 5 others (1989). IGF-II: potential autocrine/paracrine growth factor for human breast cancer acting via the IGF-I receptor. *Mol. Endocrinol.*, **3**, 1701– 1709.

PERES, R., BETHOLTZ, C., WESTERMARK, B. & HELDIN, C.-H. (1987). Frequent expression of growth factors for mesenchymal cells in human mammary carcinoma cell lines. *Cancer Res.*, **47**, 3425–3429.

PEYRAT, J.P., BONNETERRE, J., VENNIN, P.H., JAMMES, H., BENSCART, R., HEIQUET, B., DJIANE, J., LEFEBVRE, J. & DEMEILLE, A. (1990). Insulin-like growth factor 1 receptors (IGF1-R) and IGF1 in human breast tumors. *J. Steroid Biochem. Molec. Biol.*, **37**, 823–837.

PICARD, O., ROLLAND, Y. & POUPON, M.F. (1986). Fibroblast dependent tumorigenicity of cells in nude mice: implications for implantation of metastases. *Cancer Res.*, **46**, 3290–3294.

ROZENGURT, E., SINNET-SMITH, J. & TAYLOR-PAPADIMITRIOU, J. (1985). Production of PDGF-like growth factor by breast cancer cell lines. *Int. J. Cancer*, **36**, 241–252.

RYAN, M.C., JONES, D.L. & HORGAN, K. (1991). Fibroblast stimulation of human breast cancer *in vitro*. *Br. J. Cancer*, **63(XIII)**, 70.

TWENTYMAN, P.R. & LUSCOMBE, M. (1987). A study of some variables in a tetrazolium dye (MTT) based assay for cell growth and chemosensitivity. *Br. J. Cancer*, **56**, 279–285.

VAN DER BURG, B., ISBRUCKER, L., VAN SELM-MILTENBURG, A.J.P., DE LAAT, S.W. & VAN ZOELEN, E.J.J. (1990). Role of estrogen-induced insulin-like growth factors in the proliferation of human breast cancer cells. *Cancer Res.*, **50**, 7770–7774.

VAN ROOZENDAAL, C.P., VAN OOJEN, B., KLIJN, J.M.G., CLASSEN, C., EGGERMONT, A.M.M., HENZEN-LOGMANS, S.C. & FOEKENS, J.A. (1992). Stromal influences on breast cancer cell growth. *Br. J. Cancer*, **65**, 77–81.

YEE, D., CULLEN, K.J., PAIK, S., PERDUE, J.F., HAMPTON, B., SCHWARTZ, A., LIPPMAN, M.E. & ROSEN, N. (1988). Insulin-like growth factor II mRNA expression in human breast cancer. *Cancer Res.*, **48**, 6691–6696.

YEE, D., PAIK, S., LEBOVIC, G.S. & 5 others (1989). Analysis of IGF-I gene expression in malignancy: evidence for a paracrine role in human breast cancer. *Mol. Endocrinol.*, **3**, 509–517.


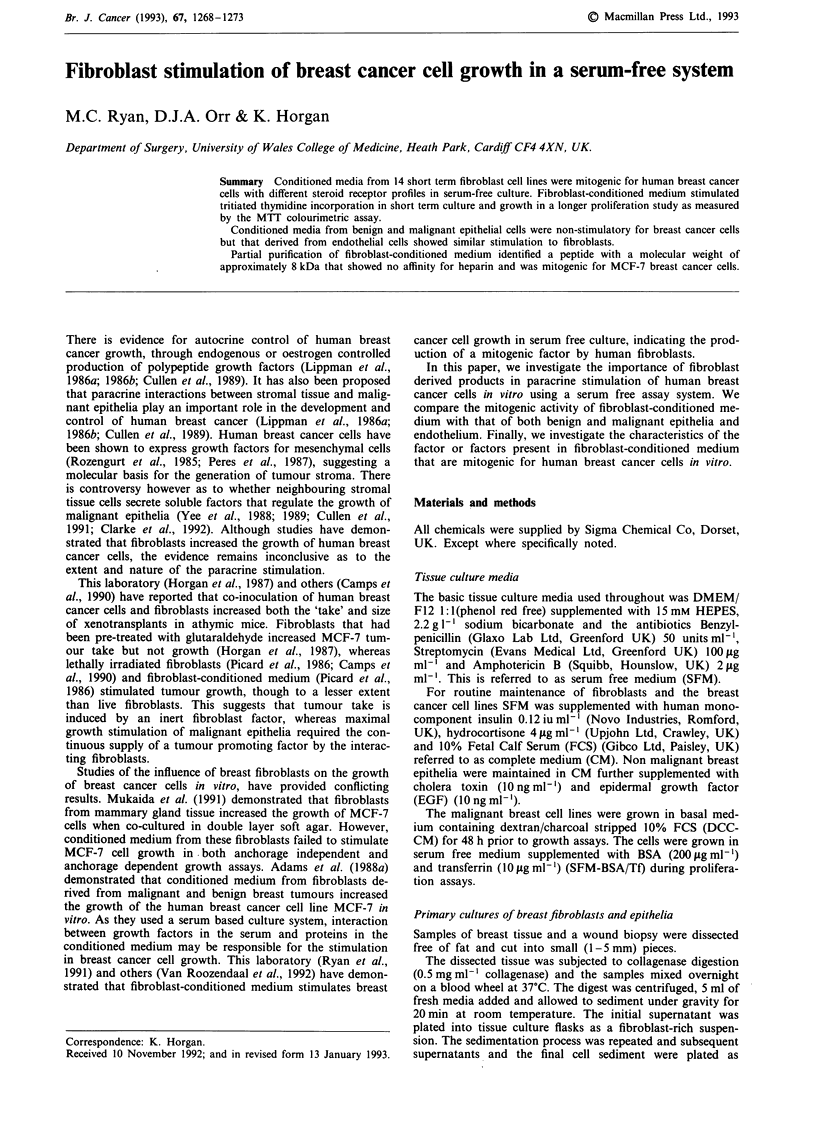

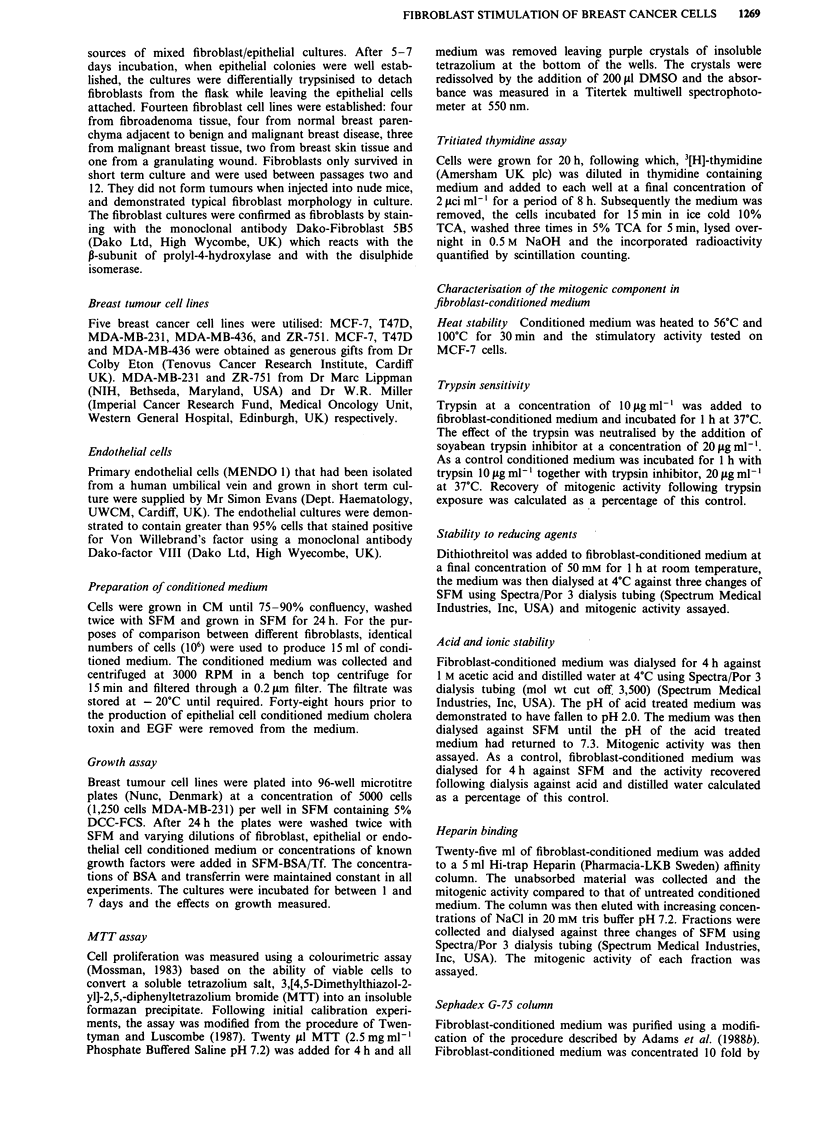

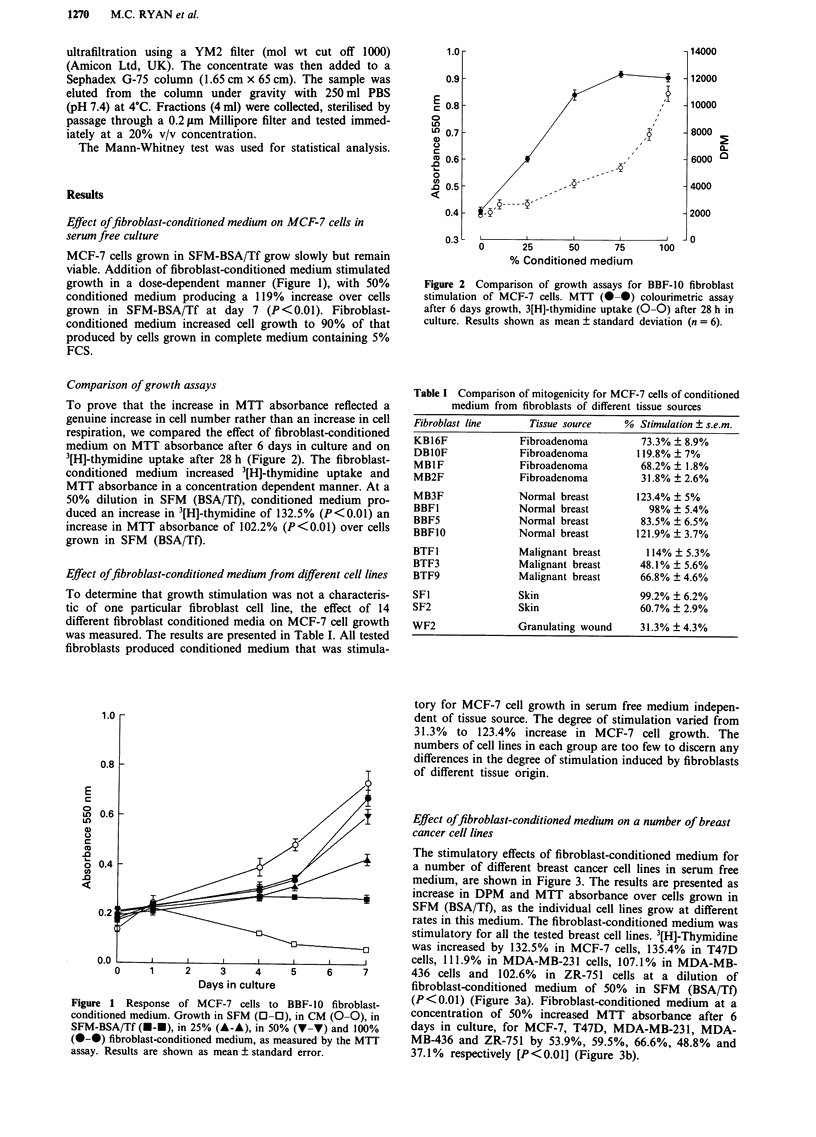

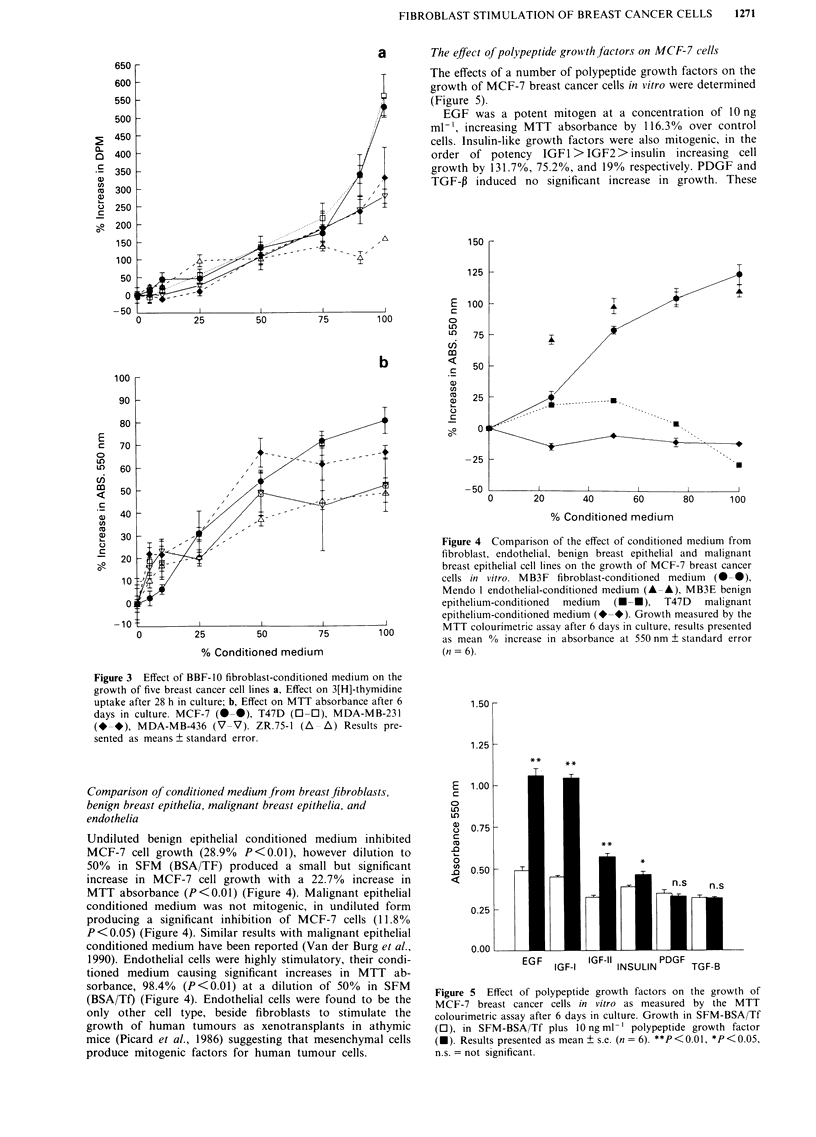

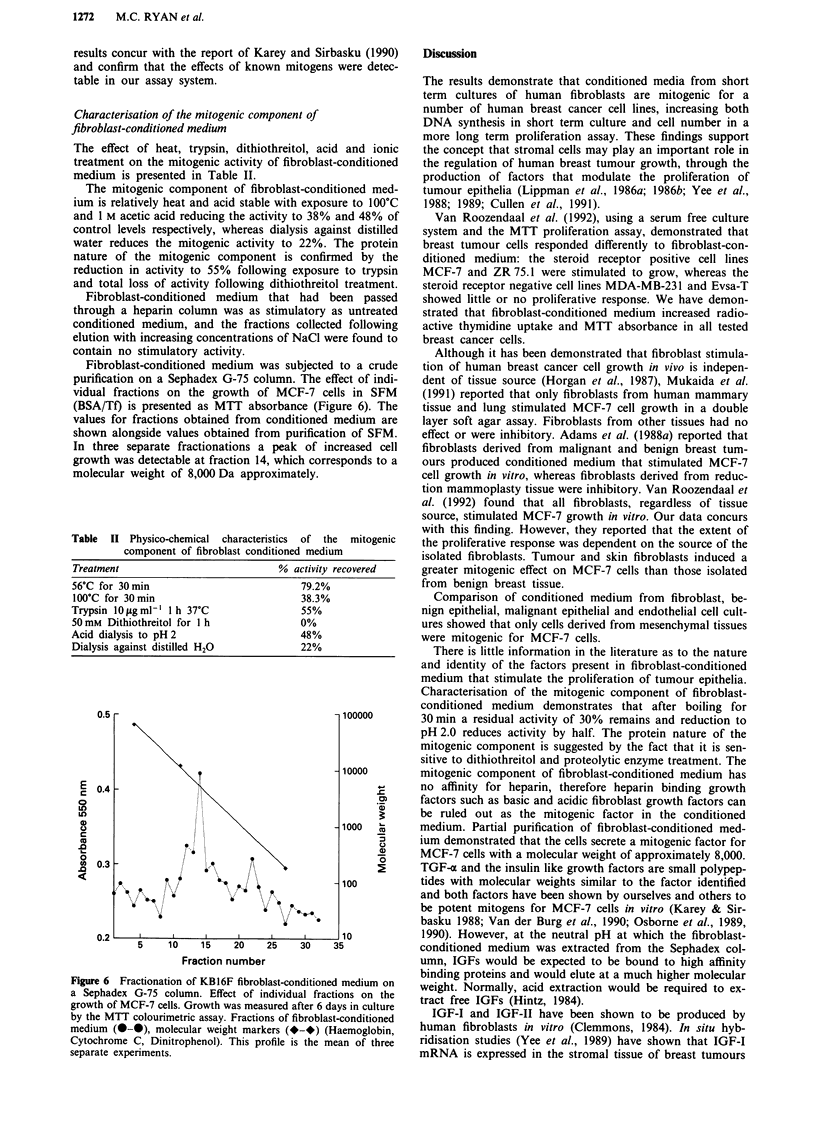

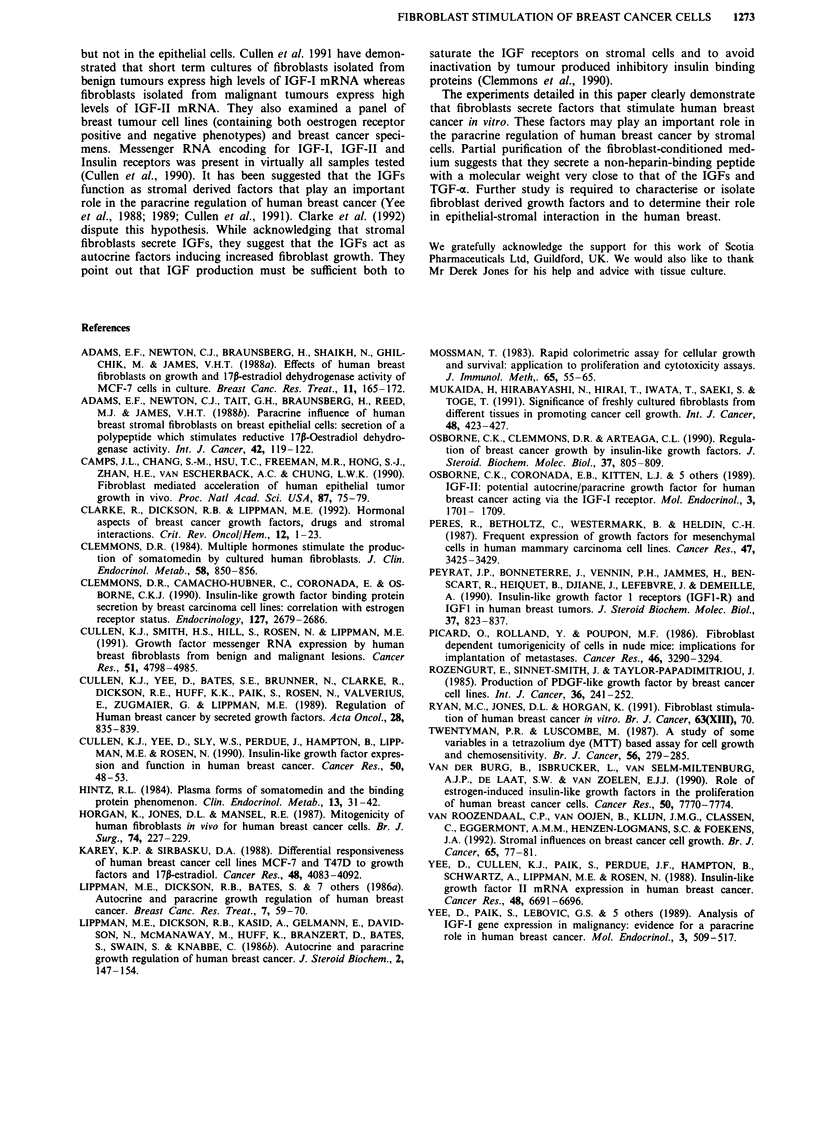


## References

[OCR_00880] Adams E. F., Newton C. J., Braunsberg H., Shaikh N., Ghilchik M., James V. H. (1988). Effects of human breast fibroblasts on growth and 17 beta-estradiol dehydrogenase activity of MCF-7 cells in culture.. Breast Cancer Res Treat.

[OCR_00883] Adams E. F., Newton C. J., Tait G. H., Braunsberg H., Reed M. J., James V. H. (1988). Paracrine influence of human breast stromal fibroblasts on breast epithelial cells: secretion of a polypeptide which stimulates reductive 17 beta-oestradiol dehydrogenase activity.. Int J Cancer.

[OCR_00890] Camps J. L., Chang S. M., Hsu T. C., Freeman M. R., Hong S. J., Zhau H. E., von Eschenbach A. C., Chung L. W. (1990). Fibroblast-mediated acceleration of human epithelial tumor growth in vivo.. Proc Natl Acad Sci U S A.

[OCR_00896] Clarke R., Dickson R. B., Lippman M. E. (1992). Hormonal aspects of breast cancer. Growth factors, drugs and stromal interactions.. Crit Rev Oncol Hematol.

[OCR_00908] Clemmons D. R., Camacho-Hubner C., Coronado E., Osborne C. K. (1990). Insulin-like growth factor binding protein secretion by breast carcinoma cell lines: correlation with estrogen receptor status.. Endocrinology.

[OCR_00901] Clemmons D. R. (1984). Multiple hormones stimulate the production of somatomedin by cultured human fibroblasts.. J Clin Endocrinol Metab.

[OCR_00912] Cullen K. J., Smith H. S., Hill S., Rosen N., Lippman M. E. (1991). Growth factor messenger RNA expression by human breast fibroblasts from benign and malignant lesions.. Cancer Res.

[OCR_00918] Cullen K. J., Yee D., Bates S. E., Brunner N., Clarke R., Dickson R. E., Huff K. K., Paik S., Rosen N., Valverius E. (1989). Regulation of human breast cancer by secreted growth factors.. Acta Oncol.

[OCR_00927] Cullen K. J., Yee D., Sly W. S., Perdue J., Hampton B., Lippman M. E., Rosen N. (1990). Insulin-like growth factor receptor expression and function in human breast cancer.. Cancer Res.

[OCR_00931] Hintz R. L. (1984). Plasma forms of somatomedin and the binding protein phenomenon.. Clin Endocrinol Metab.

[OCR_00935] Horgan K., Jones D. L., Mansel R. E. (1987). Mitogenicity of human fibroblasts in vivo for human breast cancer cells.. Br J Surg.

[OCR_00940] Karey K. P., Sirbasku D. A. (1988). Differential responsiveness of human breast cancer cell lines MCF-7 and T47D to growth factors and 17 beta-estradiol.. Cancer Res.

[OCR_00945] Lippman M. E., Dickson R. B., Bates S., Knabbe C., Huff K., Swain S., McManaway M., Bronzert D., Kasid A., Gelmann E. P. (1986). 8th San Antonio Breast Cancer Symposium--Plenary lecture. Autocrine and paracrine growth regulation of human breast cancer.. Breast Cancer Res Treat.

[OCR_00962] Mukaida H., Hirabayashi N., Hirai T., Iwata T., Saeki S., Toge T. (1991). Significance of freshly cultured fibroblasts from different tissues in promoting cancer cell growth.. Int J Cancer.

[OCR_00968] Osborne C. K., Clemmons D. R., Arteaga C. L. (1990). Regulation of breast cancer growth by insulin-like growth factors.. J Steroid Biochem Mol Biol.

[OCR_00973] Osborne C. K., Coronado E. B., Kitten L. J., Arteaga C. I., Fuqua S. A., Ramasharma K., Marshall M., Li C. H. (1989). Insulin-like growth factor-II (IGF-II): a potential autocrine/paracrine growth factor for human breast cancer acting via the IGF-I receptor.. Mol Endocrinol.

[OCR_00979] Peres R., Betsholtz C., Westermark B., Heldin C. H. (1987). Frequent expression of growth factors for mesenchymal cells in human mammary carcinoma cell lines.. Cancer Res.

[OCR_00987] Peyrat J. P., Bonneterre J., Vennin P. H., Jammes H., Beuscart R., Hecquet B., Djiane J., Lefebvre J., Demaille A. (1990). Insulin-like growth factor 1 receptors (IGF1-R) and IGF1 in human breast tumors.. J Steroid Biochem Mol Biol.

[OCR_00992] Picard O., Rolland Y., Poupon M. F. (1986). Fibroblast-dependent tumorigenicity of cells in nude mice: implication for implantation of metastases.. Cancer Res.

[OCR_00997] Rozengurt E., Sinnett-Smith J., Taylor-Papadimitriou J. (1985). Production of PDGF-like growth factor by breast cancer cell lines.. Int J Cancer.

[OCR_01005] Twentyman P. R., Luscombe M. (1987). A study of some variables in a tetrazolium dye (MTT) based assay for cell growth and chemosensitivity.. Br J Cancer.

[OCR_01022] Yee D., Cullen K. J., Paik S., Perdue J. F., Hampton B., Schwartz A., Lippman M. E., Rosen N. (1988). Insulin-like growth factor II mRNA expression in human breast cancer.. Cancer Res.

[OCR_01028] Yee D., Paik S., Lebovic G. S., Marcus R. R., Favoni R. E., Cullen K. J., Lippman M. E., Rosen N. (1989). Analysis of insulin-like growth factor I gene expression in malignancy: evidence for a paracrine role in human breast cancer.. Mol Endocrinol.

[OCR_01016] van Roozendaal C. E., van Ooijen B., Klijn J. G., Claassen C., Eggermont A. M., Henzen-Logmans S. C., Foekens J. A. (1992). Stromal influences on breast cancer cell growth.. Br J Cancer.

[OCR_01010] van der Burg B., Isbrücker L., van Selm-Miltenburg A. J., de Laat S. W., van Zoelen E. J. (1990). Role of estrogen-induced insulin-like growth factors in the proliferation of human breast cancer cells.. Cancer Res.

